# Perioperative Mortality and the Long-Term Outcome of Endovascular Abdominal Aneurysm Repair (EVAR): A Single-Centre Experience

**DOI:** 10.7759/cureus.49260

**Published:** 2023-11-22

**Authors:** Mohamed S M Elshikhawoda, Muhammad Numan Zahid, Steven H S Tan, Ahmed Hashim Ahmed Mohamed, Doaa Abdalaziz Salih Abdalaziz, Ali Yasen Y Mohamedahmed, Sohaib Jararaa, Mahmoud Okaz, Abdelrhman Elsanosi, Hassan Jararah

**Affiliations:** 1 Vascular and Endovascular Surgery, Glan Clwyd Hospital, Rhyl, GBR; 2 Vascular and Endovascular surgery, Glan Clwyd Hospital, Rhyl, GBR; 3 Surgery, University Hospital of Wales, Cardiff, GBR; 4 General Surgery, Faculty of Medicine, The National Ribat University, Khartoum, SDN; 5 General Surgery, University Hospital of North Tees, Stock-on-Tees, GBR; 6 General Surgery, The Royal Wolverhampton National Health Service (NHS) trust, Wolverhampton, GBR; 7 Vascular Surgery, Glan Clwyd Hospital, Rhyl, GBR; 8 Vascular Surgery, University Hospital Hairmyres, Scotland, GBR

**Keywords:** endovascular aortic aneurysm repair, endo-leak, evar complications, perioperative mortality, endovascular aortic repair, abdominal aortic aneurysm

## Abstract

Background

Abdominal aortic aneurysm (AAA) is a dangerous disorder characterised by abnormal enlargement of the abdominal aorta. The severity of the aneurysm and the presence of symptoms determine the necessary monitoring or treatment to prevent potential fatalities. The objective of this study is to estimate the perioperative mortality and long-term outcome of endovascular abdominal aneurysm repair (EVAR).

Patients and methods

This is a descriptive, retrospective, observational study. We retrieved the data of the AAA patients who underwent EVAR at Glan Clwyd Hospital from January 2015 to January 2023. The study sample consisted of patients diagnosed with isolated AAA, with or without iliac branch involvement, who were deemed suitable for EVAR based on factors such as advanced age, presence of comorbidities, the complexity of the condition, history of prior surgery, fulfillment of indication criteria, and patient desire. The data was analysed using SPSS statistical software, version 21.0 (IBM Corp., Armonk, NY).

Results

Two hundred and twenty-two patients were studied. The outcome of the EVAR among the patients was endo-leak 28.4% (n = 63); migration 1.4% (n = 3); blockage 0.5% (n = 1); infolding 0.5% (n = 1); perioperative mortality 1.4% (3); and other complications like access site or acute kidney injury were 1.4% (n = 3). However, no complications were reported in most of the patients, 66.7% (n = 148). Upon evaluating the variables that could affect the outcome, we observed that the ASA grade, comorbidities, and the indication of the intervention had a significant effect on the outcome (P values = 0.000, 0.048, and 0.014, respectively).

Conclusion

The findings demonstrate that when EVAR is performed by a skilled team adhering to proper criteria, the results are optimal. The mortality rate during the perioperative period was 1.4%. Furthermore, we have shown a satisfactory rate of complications when compared to international data.

## Introduction

Abdominal aortic aneurysm (AAA) is a dangerous disorder characterised by abnormal enlargement of the abdominal aorta. The severity of the aneurysm and the presence of symptoms determine the necessary monitoring or treatment to prevent potential fatalities. AAA may be discovered either unintentionally or during a rupture. An arterial aneurysm is defined as a long-lasting localised widening of a blood vessel that is at least 150% larger than the nearby normal diameter of that artery [1].

Prior to the 1990s, the only available method for treating AAA patients was open surgical repair. However, in 1986, the first study on endovascular stent graft-based repair was published, which paved the way for the development of minimally invasive endovascular aneurysm repair (EVAR). Subsequently, the investigations began to concentrate on this endovascular technique [2-4]. Given recent advances in device technology and its ability to address anatomical challenges, EVAR has been the preferred alternative in the last 20 years [5,6].

Definitions

Symptomatic unruptured AAA refers to the presence of pain or tenderness upon palpation, specifically localised to the AAA or extending to the back, whether or not a pulsatile mass is detected on physical examination.

Endoleak is a complication that arises after endovascular aneurysm repair (EVAR) and involves the leakage of blood into the aneurysm sac following the implantation of a stent graft. Endoleaks can be categorised into five distinct types. Type I: Leak at the graft attachment site (Ia: Proximal, Ib: Distal); Type II (most common): aneurysm sac filling via branch vessel (IIa: single vessel, IIb: Multiple vessels); Type III: Leak through a defect in the graft; Type IV: Leaks through graft fabric as a result of graft porosity, often intraoperative, and resolve with cessation of anticoagulants; and Type V: continued expansion of the aneurysm sac without demonstrable leak on imaging (endotension).

## Materials and methods

Study design and population

The present study is a descriptive, retrospective, observational study. The study sample consisted of patients diagnosed with isolated infrarenal AAA, with or without iliac branch involvement, who were deemed suitable for EVAR based on factors such as advanced age, presence of comorbidities, the complexity of the condition, history of prior surgery, fulfillment of indication criteria, and patient desire. Furthermore, our exclusion criteria encompassed patients who had insufficient data or were not accessible for additional follow-up via in-person visits or phone conversations.

Data collection

We retrieved the data of the AAA patients who underwent EVAR at Glan Clwyd Hospital between January 2015 and January 2023. The data were obtained from Glan Clwyd Hospital's databases and supplemented with the patient's medical records. The baseline characteristics considered for this study included demographic and clinical factors such as age, gender, cardiovascular risk factors (hypertension, diabetes mellitus, and smoking), history of coronary artery disease, chronic obstructive pulmonary disease, congestive cardiac failure, stroke, peripheral vascular disease, and chronic kidney disease. Additionally, the American Society of Anaesthesiologists' (ASA's) grade was taken into account. These characteristics were evaluated according to the reporting standards for infrared AAA repair.

All patients undergo a rigorous evaluation process, which includes gathering their medical history, doing physical examinations, and performing comprehensive blood tests, including prohormone of brain natriuretic peptide (proBNP) and troponin. Additionally, stress tests are conducted in the cardiac electrophysiology department. The findings from these assessments were then discussed by the multidisciplinary team (MDT) to determine the most suitable treatment approach for each patient.

Follow-up

Post-hospitalization, the patients were closely followed. To monitor progress following the treatment, a multislice CT scan angiography was conducted one month later and subsequently on an annual basis. The purpose was to evaluate the stent graft and any alterations in the size of the aneurysm. Abdominal ultrasonography was performed on patients who had hypersensitivity to contrast medium or high serum creatinine levels. If the patient did not come to their scheduled follow-up visits, we took the initiative to contact them by phone and carefully recorded any possible incidents or challenges. All patients were followed for a minimum of 10 to 48 months to assess sac size and any potential complications or adverse events.

Data analysis

The data was analysed using SPSS statistical software, version 21.0 (IBM Corp., Armonk, NY). Categorical demographic and clinical characteristics were described as frequency (percentage). In addition, customarily distributed quantitative characteristics were shown as the mean with standard deviation. We conducted a chi-square test to evaluate the potential factors influencing the outcome. A P value less than 0.05 was deemed statistically significant.

## Results

We enrolled a total of 222 patients. The majority of the patients were male, accounting for 89.6% (n = 199), and had a mean age of 76.7 ± 6.5 standard deviation. The majority of the patients were former smokers and had additional medical conditions, including hypertension (67.6%), ischemic heart disease (32.4%), chronic obstructive pulmonary disease (25.7%), and diabetes (21.2%). Nevertheless, the remaining comorbidities were less common (Table 1). The patient's anaesthetic assessment revealed that the majority of them had an ASA grade of 3 (Table 1).

**Table 1:** Patient demographics and comorbidities ASA: American Society of Anesthesiologists

		Number	Percentage
Gender	Male	199	89.6%
	Female	23	10.4%
ASA	1	2	0.9%
	2	53	23.9%
	3	155	69.8%
	4	11	5%
	5	1	0.5%
Smoking	Current smoker	41	18.5%
	Ex-smoker	121	54.5%
	Never smoked	60	27%
Diabetes mellitus	Yes	47	21.2%
	No	175	78.8%
Hypertension	Yes	150	67.6%
	No	72	32.4
Chronic obstructive pulmonary diseases	Yes	57	25.7%
	No	165	74.3%
Ischemic Heart Diseases	Yes	72	32.4
	No	150	67.6
Congestive cardiac failure	Yes	10	4.5%
	No	212	95.5%
Chronic kidney disease	Yes	26	11.7
	No	196	88.3%
Stroke	Yes	13	5.9%
	No	209	94.1%
Peripheral vascular disease	Yes	15	6.8%
	No	207	93.2%
Total		222	100%

The national surveillance of AAAs of size more than or equal to 5.5 cm was the most frequently recoded indication for repair, and the rest of the indications were less frequent (Table 2).

**Table 2:** Indications of AAA Repair AAA: Abdominal Aortic Aneurysm; NAAASP: National Abdominal Aortic Aneurysm Surveillance Program

Indication	Number	Percentage
≥55mm screen detected aneurysm (NAAASP)	91	41%
≥55mm screen detected aneurysm (non-NAAASP)	35	15.8%
≥55mm lesion non-screen detected aneurysm	58	26.1%
Symptomatic	12	5.4%
Rapid growth	6	2.7%
Other threshold	5	2.3%
Iliac Aneurysm	15	6.8%
Total	222	100%

The preoperative mean diameter of AAA was 61.5 ± 10.8 mm standard deviation (SD). The follow-up AAA sac diameter after EVAR was 61.1 ± 10 mm SD at 1 month, 58.7 ± 15 mm SD at 12 months, 62.9 ± 14.5 mm SD at 24 months, 67.2 ± 8.9 mm SD at 36 months, and 57.5 ± 18.1 mm SD at 48 months.

The outcome of the EVAR among the patients was endoleak 28.4% (n = 63); migration 1.4% (n = 3); blockage 0.5% (n = 1); infolding 0.5% (n = 1); perioperative mortality 1.4% (n = 3); and other complications such as access site or acute kidney injury were 1.4% (n = 3). However, no complications were reported in most of the patients, 66.7% (n = 148). Upon evaluating the variables that could affect the outcome, we observed that the ASA grade, comorbidities, and the indication of the intervention had a significant effect on the outcome (P values = 0.000, 0.048, and 0.014, respectively) (Table 3).

**Table 3:** Factors affecting the outcome NAAASP: National Abdominal Aortic Aneurysm Screening Programme

	Factors	Outcome							
		No complications	Endoleak	Migration	Blockage	Others	Death	In folding	P Value
ASA	1	2 (0.9%)	0	0	0	0	0	0	
	2	42 (18.9%)	8 (3.6%)	1 (0.4%)	1(0.4%)	1(0.4%)	0	0	
	3	97 (43.9%)	52(24.6%)	1(0.4%)	0	0	2(0.9%)	1	0.000
	4	7 (3.1%)	3 (1.3%)	1(0.4%)	0	0	0	0	
	5	0	0	0	0	0	1(0.4%)	0	
Comorbidities	yes	27(12.1%)	59(26.5%)	1(0.4%)	1(0.4%)	3(1.3%)	3(1.3%)	1(0.4%)	0.048
	No	121(54.9 %)	4(1.8%)	2(0.9%)	0	0	0	0	
Indication for Intervention	≥55mm screen detected aneurysm (NAAASP)	55 (24.7%)	32(14.4%)	1(0.4%)	1 (0.4%)	2 (0.9%)	0	0	
	≥55mm screen detected aneurysm (non-NAAASP)	28 (12.6%)	9 (4%)	0	0	0	0	0	0.014
	≥55mm lesion non-screen detected aneurysm	40 (18%)	15 (6.8%)	0	0	1(0.4%)	2(0.9%)	0	
	Symptomatic	8 (3.6%)	2 (0.9%)	1 (0.4%)	0	0	0	1(0.4%)	
	Rapid growth	3 (1.3%)	1(0.4%)	1(0.4%)	0	0	1(0.4%)	0	
	Other thresholds	5 (2.5%)	0	0	0	0	0	0	
	Iliac Aneurysm	11(4.8%)	4 (1.8%)	0	0	0	0	0	

Regarding the endoleak, Type II was the most frequently reported in 57 (90.4%) patients, followed by Type I in 5 (7.9%) patients, and Type 3 (1.7%) in one patient. However, most of them were treated conservatively (Table 4). The mean length of hospital stay was 2.4 ± 1 day SD.

**Table 4:** Endoleak management

Intervention	Number	Percentage
Not applicable	159	71.4%
Observation	55	24.8%
Endoanchor	2	0.9%
Coiling	1	0.5%
Onyx injection	1	0.5%
Open repair	2	0.9%
Cuff extension	1	0.5%
Cuff insertion and endoanchor	1	0.5%
Total	222	100%

## Discussion

This study establishes that endovascular repair offers a viable substitute for open repair in the management of AAA. Furthermore, apart from a decrease in 30-day mortality, our findings indicate that EVAR is linked to a reduced occurrence of other negative outcomes, such as acute renal failure, ischemic colitis, the need for additional surgeries, and the length of hospital stays.

Examinations of national databases indicate that in the last 20 years, EVAR has become more popular than open AAA repair [4-7]. The simplicity of using EVAR has resulted in a higher rate of AAA repair among older individuals [4-7]. EVAR is a favourable choice for older individuals with serious additional medical conditions, for whom open surgery poses an excessively high level of risk [8]. Among the elderly, utilising an endovascular method has more favourable results, with perioperative mortality ranging from 0 to 6%. This aligns with our study, which showed perioperative mortality of 1.4% [9-12].

Complications such as access site complications, acute kidney injury, and graft thrombosis are less frequently reported in the literature [13-16]. Our study showed that all these complications were reported in 3.8% of our patients, which agrees with the former studies. According to multiple studies, Type II endoleak is the most prevalent form of endoleak, with reported rates of up to 25%. [17,18]. The OVER trial documented a total occurrence rate of endoleaks at 30.5%, with Type I endoleaks accounting for 12.3%, Type II endoleaks accounting for 75.9%, Type III endoleaks accounting for 3.2%, Type IV endoleaks accounting for 2.7%, and Type V endoleaks accounting for 5.9% [11]. This is not exactly similar to our study; this might be related to the patient numbers and the levels of the centres.

We treated most of our patients with Type II endoleak conservatively; in contrast, we treated Types I and III promptly due to the rapid increase in sac size with either cuff extension, endoanchor, or conversion to open. Moreover, we treated two patients with Type II by coiling and onyx injection. The vast majority of the authors agreed on conservative management for Type II endoleak as a safe option [19,20]. Additionally, Moulakakis et al. have suggested that open intervention is superior to endovascular in treating endoleak [21].

When assessing the factors that may impact the outcome, we noticed that the ASA grade and comorbidities, which are related to each other, have a significant impact on the outcome. Furthermore, the indication of AAA repair was particularly noteworthy, particularly in patients with endoleaks that may be associated with the iliac aneurysm or the size of the aneurysm.

Study limitations

Although our centre is a high-capacity facility in the UK for performing EVAR, the single-center nature of this study limits its applicability. Furthermore, one of the limitations of our study was that we gathered data retrospectively. Consequently, we could only analyse the data that was already included in the patient's files.

## Conclusions

This study examined the outcomes of endovascular aneurysm repair (EVAR) patients at our medical facility. The findings demonstrate that when EVAR is performed by a skilled team and in accordance with proper criteria, it can lead to optimal results. The mortality rate during the perioperative period was 1.4%. In addition, we have demonstrated a reasonable rate of complications in comparison to other national and international facilities.
